# Long-Term Tubular Dysfunction in Childhood Cancer Survivors; DCCSS-LATER 2 Renal Study

**DOI:** 10.3390/cancers14112754

**Published:** 2022-06-01

**Authors:** Esmee C. M. Kooijmans, Helena J. H. van der Pal, Saskia M. F. Pluijm, Margriet van der Heiden-van der Loo, Leontien C. M. Kremer, Dorine Bresters, Eline van Dulmen-den Broeder, Marry M. van den Heuvel-Eibrink, Jacqueline J. Loonen, Marloes Louwerens, Sebastian J. C. Neggers, Cécile Ronckers, Wim J. E. Tissing, Andrica C. H. de Vries, Gertjan J. L. Kaspers, Arend Bökenkamp, Margreet A. Veening, on behalf of the Dutch LATER Study Group

**Affiliations:** 1 Department of Pediatric Oncology, Emma Children’s Hospital, Amsterdam UMC, Vrije Universiteit Amsterdam, 1081 HV Amsterdam, The Netherlands; e.vandulmen-denbroeder@amsterdamumc.nl (E.v.D.-d.B.); g.j.l.kaspers@prinsesmaximacentrum.nl (G.J.L.K.); ma.veening@prinsesmaximacentrum.nl (M.A.V.); 2 Princess Máxima Center for Pediatric Oncology, 3584 CS Utrecht, The Netherlands; h.j.h.vanderpal@prinsesmaximacentrum.nl (H.J.H.v.d.P.); s.m.f.pluijm@prinsesmaximacentrum.nl (S.M.F.P.); m.vanderheiden@prinsesmaximacentrum.nl (M.v.d.H.-v.d.L.); l.c.m.kremer@prinsesmaximacentrum.nl (L.C.M.K.); d.bresters@prinsesmaximacentrum.nl (D.B.); m.m.vandenheuvel-eibrink@prinsesmaximacentrum.nl (M.M.v.d.H.-E.); cecile.ronckers@mhb-fontane.de (C.R.); w.j.e.tissing@prinsesmaximacentrum.nl (W.J.E.T.); a.c.h.devries@erasmusmc.nl (A.C.H.d.V.); 3 Dutch Childhood Oncology Group, 3584 CS Utrecht, The Netherlands; 4 Wilhelmina Children’s Hospital, University Medical Center Utrecht, 3584 EA Utrecht, The Netherlands; 5 Deparmtnet of Pediatric Oncology, Emma Children’s Hospital, Amsterdam UMC, University of Amsterdam, 1105 AZ Amsterdam, The Netherlands; 6 Willem Alexander Children’s Hospital, Leiden University Medical Center, 2333 ZA Leiden, The Netherlands; 7 Department of Pediatric Oncology, Sophia Children’s Hospital, Erasmus Medical Center, 3015 GD Rotterdam, The Netherlands; 8 Department of Hematology, Radboud University Medical Center, 6525 GA Nijmegen, The Netherlands; jacqueline.loonen@radboudumc.nl; 9 Department of Internal Medicine, Leiden University Medical Center, 2333 ZA Leiden, The Netherlands; m.louwerens@lumc.nl; 10 Department of Internal Medicine, Erasmus Medical Center, 3015 GD Rotterdam, The Netherlands; s.j.c.neggers@prinsesmaximacentrum.nl; 11 Department of Pediatric Oncology, University of Groningen, University Medical Center Groningen, 8713 GZ Groningen, The Netherlands; 12 Department of Pediatric Nephrology, Emma Children’s Hospital, Amsterdam UMC, Vrije Universiteit Amsterdam, 1081 HV Amsterdam, The Netherlands; a.bokenkamp@amsterdamumc.nl

**Keywords:** childhood cancer survivor, nephrotoxicity, tubular dysfunction

## Abstract

**Simple Summary:**

We studied survivors of childhood cancer who received cancer treatment that might affect the kidneys and compared them to controls from the general population. We investigated if there was a difference in the occurrence of tubular dysfunction. The tubules are the part of the kidney responsible for reabsorption of needed substances to the blood and the removal of wastes. After around 25 years since their cancer diagnosis, we found that in general there were no differences between survivors and controls, but survivors more often had losses of small proteins in the urine. Yet, some survivors of childhood cancer were found to have an increased risk of tubular dysfunction. Namely, survivors treated with the chemotherapeutic agents ifosfamide, cisplatin or carboplatin. Therefore, these patients should be monitored during their follow-up.

**Abstract:**

The aim of this nationwide cross-sectional cohort study was to determine the prevalence of and risk factors for tubular dysfunction in childhood cancer survivors (CCS). In the DCCSS-LATER 2 Renal study, 1024 CCS (≥5 years after diagnosis), aged ≥ 18 years at study, treated between 1963 and 2001 with potentially nephrotoxic therapy (i.e., nephrectomy, abdominal radiotherapy, total body irradiation, cisplatin, carboplatin, ifosfamide, high-dose cyclophosphamide, or hematopoietic stem cell transplantation) participated, and 500 age- and sex-matched participants from Lifelines acted as controls. Tubular electrolyte loss was defined as low serum levels (magnesium < 0.7 mmol/L, phosphate < 0.7 mmol/L and potassium < 3.6 mmol/L) with increased renal excretion or supplementation. A α1-microglobulin:creatinine ratio > 1.7 mg/mmol was considered as low-molecular weight proteinuria (LMWP). Multivariable risk analyses were performed. After median 25.5 years follow-up, overall prevalence of electrolyte losses in CCS (magnesium 5.6%, potassium 4.5%, phosphate 5.5%) was not higher compared to controls. LMWP was more prevalent (CCS 20.1% versus controls 0.4%). LMWP and magnesium loss were associated with glomerular dysfunction. Ifosfamide was associated with potassium loss, phosphate loss (with cumulative dose > 42 g/m^2^) and LMWP. Cisplatin was associated with magnesium loss and a cumulative dose > 500 mg/m^2^ with potassium and phosphate loss. Carboplatin cumulative dose > 2800 mg/m^2^ was associated with potassium loss. In conclusion, long-term tubular dysfunction is infrequent. Yet, ifosfamide, cisplatin and carboplatin are risk factors.

## 1. Introduction

As a result of improved survival rates, currently eight out of ten children diagnosed with cancer will survive five or more years after diagnosis [1]. An effect of this increased survival is the manifestation of late effects [2].

A well-known late effect is nephrotoxicity, manifesting as glomerular and/or tubular damage. This can be caused by chemotherapy, including cisplatin, carboplatin, ifosfamide, cyclophosphamide, radiation to the kidney area or nephrectomy [3,4].

Tubular damage is characterized by electrolyte derangements and urinary wasting of low molecular weight (LMW) proteins. Prolonged hypophosphatemia may lead to hypophosphatemic rickets in children [5] with the consequence of growth impairment [6] or osteomalacia in adults [7]. The clinical impact of other persistent electrolyte alterations is less apparent.

Research among childhood cancer survivors (CCS) has shown an association of platinum compounds and ifosfamide exposure with tubular injury [4,8,9,10,11,12]. Although screening guidelines for CCS often also advise tubular dysfunction screening for other potentially nephrotoxic therapies, including nephrectomy and radiotherapy to the renal area [13,14], no clear associations of these modalities have been described in the literature.

Studies assessing tubular toxicity in CCS are limited and are often hampered by small patient numbers, limiting good risk factor analyses [3]. In addition, recent longitudinal studies up to 10 years follow-up suggest that tubular function may improve over the years [10]. Still, very long-term (>20 years) follow-up studies have not yet been performed. As ifosfamide and platinum compounds are still widely used in the treatment of several childhood malignancies [15,16,17], it is important to gain more knowledge of the effects on the very long term.

The aim of this nationwide multicenter cross-sectional cohort study was to evaluate the prevalence of and risk factors for tubular dysfunction in very long-term CCS in comparison with matched controls.

## 2. Materials and Methods

### 2.1. Study Population

For the Dutch Childhood Cancer Survivor Study (DCCSS) LATER cohort part 2 study, CCS diagnosed at the age of 0 to 17 years, treated between 1963–2001 in one of the childhood cancer centers in the Netherlands and with a survival of at least 5 years from diagnosis were eligible.

Additional inclusion criteria for this sub-study on nephrotoxicity were: (1) age ≥ 18 years at the time of the study, (2) sufficient understanding of the Dutch language to provide informed consent, and (3) treatment with potentially nephrotoxic treatment, i.e., (a) nephrectomy (unilateral, partial bilateral), (b) radiotherapy involving one or both kidneys in the field (abdominal, total body irradiation (TBI), in nephrectomized patients radiotherapy in the field of the remnant kidney), (c) chemotherapy: cisplatin, carboplatin, ifosfamide or high-dose (HD)-cyclophosphamide ≥ 1 g/m^2^ per course or ≥10 g/m^2^ in total [18,19], or (d) allogeneic hematopoietic stem cell transplantation (HSCT). For HD-cyclophosphamide, information regarding dose per course was incomplete. If the cyclophosphamide cumulative dose was <10 g/m^2^, CCS were only selected if they had been treated according to the ALL7 or ALL8 protocol [20,21]. Exclusion criteria were pregnancy at time of study or a history of kidney transplantation. Three subsets have been described previously [18,19,22].

### 2.2. Controls

Lifelines is a multi-disciplinary prospective population-based cohort study examining the health and health-related behavior of 167,729 persons living in the north of the Netherlands in a unique three-generation design [23]. First, via participating general practitioners, an index population aged 25–49 years was recruited. Second, older and younger family members were invited to participate. Last, adults could self-register on the Lifelines website to take part. The inclusion period was between 2006 and 2013, but most participants (57%) were included in the last two years. Lifelines employs a broad range of investigative procedures to assess biomedical, socio-demographic, behavioral, physical and psychological factors which contribute to the health and disease of the general population, with a special focus on multi-morbidity and complex genetics [23,24].

A total of 500 controls of Lifelines were included. The same exclusion applied as for CCS, with the additional exclusion criterion of a history of cancer. Controls were randomly selected and matched to CCS by age and sex using frequency matching.

### 2.3. Data Collection

Details on the diagnosis and treatment of primary malignancy and any recurrences are stored in a central database for all CCS, with the exception of survivors refusing storage of their data. Treatment details include cumulative doses of chemotherapy, radiation field and fractionation schedule and types of surgery. At the time of the study, blood and urine laboratory tests were performed, and a physical examination was conducted. Patients received questionnaires about their medical history and lifestyle. Study visits took place between October 2016 and February 2020. This study was approved by the Institutional Review Board of Emma Children’s hospital of the Amsterdam University Medical Centers (NL35046.018.11). Written informed consent was obtained from all participants.

From the controls, we collected demographic data, and results of questionnaires, a physical examination and laboratory testing. For both CCS and controls (fasting) blood and urine samples were collected in the morning on the same day. Urine was stored at −80 °C. For CCS, laboratory tests were performed locally in the participating centers, except for alpha-1-microglobulin which was determined in one central laboratory. For controls, all tests were performed in one clinical laboratory. In both CCS and controls, all electrolytes were measured on a routine chemistry platform; phosphate was measured using a molybdate UV assay (Cobas8000, Roche, Mannheim, Germany in controls and Cobas6000, Rokreuz, Switzerland in CCS), magnesium was measured by the xylidyl blue method using a colorimetric assay (Cobas8000, Roche, Mannheim, Germany in controls and Cobas6000, Rokreuz, Switzerland in CCS) and potassium was measured via an indirect ISE module (Cobas8000, Roche, Mannheim, Germany in controls and CCS).

### 2.4. Definition of Tubular Dysfunction

Tubular function was evaluated based on tubular electrolyte loss, low-molecular weight proteinuria (LMWP) and metabolic acidosis.

Tubular electrolyte loss was defined as low serum levels in combination with increased renal excretion or use of electrolyte supplementation in the absence of underfeeding. Moreover, serum magnesium < 0.70 mmol/L was defined as hypomagnesemia, serum potassium < 3.6 mmol/L as hypokalemia and serum phosphate < 0.70 mmol/L as hypophosphatemia [25]. In case of low serum levels, the fractional excretion was calculated to distinguish renal from non-renal causes. Fractional magnesium excretion > 2% was considered of renal origin and calculated as follows [26]:

(1)  ((urine magnesium× serum creatinine)/(serum magnesium × urine creatinine ×0.7))×100

The formula used for fractional potassium excretion was

((urine potassium × serum creatinine)/(serum potassium × urine creatinine)) × 100,(2)

and it was considered indicative of tubular losses if >6.5% [27]. For hypophosphatemia, the tubular phosphate threshold (TmP/GFR) was determined based on tubular reabsorption of phosphate (TRP). TRP was calculated by

1 − ((urine phosphate/serum phosphate) × (serum phosphate/urine phosphate)).(3)

If TRP was ≤0.86, TmP/GFR was calculated as

(4) TRP × serum phosphate.

If TRP was >0.86, TmP/GFR was calculated as [28]

serum phosphate × 0.3 × TRP/(1 − 0.8 × TRP).(5)

Reference values for TmP/GFR are shown in Table 1.

Alpha-1-microglobulin (α1MG) is an LMW-protein that freely passes the glomerular membrane and is fully reabsorbed in the tubules. A value of >1.7 mg/mmol α1MG in the urine after indexing with urine creatinine was defined as LMWP [29].

A bicarbonate level < 22 mmol/L or bicarbonate or citrate supplementation was considered as metabolic acidosis and assumed to be of renal origin. For controls, no bicarbonate levels were available.

Lastly, blood samples were taken in a fasting state in 911 CCS (91%) and 494 controls (99%), *p* < 0.001.

### 2.5. Definition of Glomerular Dysfunction

The relation of tubular outcomes with glomerular function was evaluated. Glomerular filtration rate (GFR) was estimated with the creatinine and cystatin C-based Chronic Kidney Disease Epidemiology Collaboration (CKD-EPI) 2012 equation [30]. The GFR categories according to the Kidney Disease: Improving Global Outcomes (KDIGO) 2012 guidelines were assessed [31]: G1, eGFR ≥ 90 mL/min/1.73 m^2^; G2, eGFR 60–89 mL/min/1.73 m^2^; G3a, eGFR 45–59 mL/min/1.73 m^2^; G3b, eGFR 30–44 mL/min/1.73 m^2^; G4, eGFR 15–29 mL/min/1.73 m^2^; and G5, eGFR < 15 mL/min/1.73 m^2^. In addition, albuminuria was assessed using a urinary albumin-to-creatinine ratio (ACR) [31]: A1, albumin-to-creatinine ratio (ACR) < 3 mg/mmol normal; A2, ACR 3–30 mg/mmol moderately increased (i.e., microalbuminuria); and A3, ACR > 30 mg/mmol severely increased (i.e., macroalbuminuria).

### 2.6. Statistical Analyses

Descriptive statistics were used to summarize demographic and treatment variables, to compare tubular outcomes between CCS and controls and to evaluate tubular outcomes with glomerular function among CCS and controls. For comparison of continuous variables, a *t*-test, or the Mann–Whitney U test in case of non-normal distribution, was used. Nominal variables were compared using a chi-squared test or the Fisher exact test (if the number of cases in one cell was less than 5).

Risk factors for tubular dysfunction were assessed using multivariable logistic regression analyses in two ways. First, analyses with controls as reference were performed for mutually exclusive treatment groups as well as for different malignancy types. Age at study, sex, GFR and ACR were evaluated as possible confounders.

Second, the impact of individual agents was also assessed using multivariable risk models. Risk factors included exposure (yes/no) to cisplatin, carboplatin, ifosfamide, HD-cyclophosphamide, abdominal radiotherapy, TBI, (partial) nephrectomy and HSCT. Possible confounders included age at diagnosis, follow-up duration, sex, GFR and ACR. For renal potassium loss, the use of angiotensin-converting-enzyme inhibitors or angiotensin receptor blockers (yes/no) and diuretics (yes/no) were also assessed as confounders. Correlation between variables was assessed using Spearman’s rank correlation. In case the correlation coefficient between two variables was >0.6, one of the variables was excluded for the final model based on lowest prevalence or clinical consideration. Because TBI and HSCT were strongly correlated (correlation coefficient 0.77), HSCT was not included in the models. Confounders that were not significantly associated with the outcome were removed unless they caused a ≥10% change in the odds ratio (OR) of a variable included in the model. Likewise, an extra model was created in which treatment agents with at least 10 exposed cases were categorized according to cumulative dose tertiles, and *p*-values < 0.05 were considered statistically significant. Analyses were performed using IBM SPSS Statistics 25.0 (IBM Corp., Foster City, CA, USA).

## 3. Results

### 3.1. Study Population

**Figure 1:**
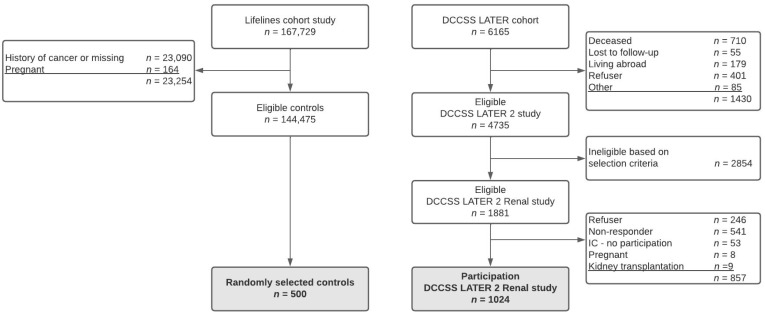
Flowchart study cohort. Abbreviations: DCCSS, Dutch Childhood Cancer Survivor Study; IC, informed consent.

The final study cohort included 1024 participants (Figure 1). The most frequent malignancies in the study population were leukemia (31.0%) and Wilms tumors (24.8%) (Table 2). The potentially nephrotoxic treatments most frequently prescribed were ifosfamide (29.3%) and HD-cyclophosphamide (27.2%), and nephrectomy was performed in 25.8%. The median age at diagnosis was 4.7 years (interquartile range (IQR) 2.4–9.2) and at study 32.5 years (IQR 27.7–38.0) with a median follow-up time of 25.5 years (IQR 21.4–30.3).

### 3.2. Prevalence of Tubular Dysfunction

The overall prevalence of tubular outcomes is shown in Table 3.

The overall prevalence of magnesium loss was not significantly different between CCS (5.6%) and controls (5.0%). Out of 5.6% of CCS with magnesium loss, 2.0% needed supplementation, while none of the controls did. In addition, CCS more often had severe hypomagnesemia < 0.6 mmol/L compared to controls (respectively, 25/1003 (2.5%) in 10 out of 25 despite supplementation, and 1/500 (0.2%), *p* < 0.001).

Tubular potassium loss was comparable in CCS (4.5%) and controls (4.0%). Out of 4.5% of CCS, nine (0.9%) were taking potassium supplementation, and no controls did.

Tubular phosphate loss was less prevalent in CCS (5.5%) compared to controls (10.8%). Still, three CCS (0.3%) were receiving phosphate supplementation, while none of the controls did. Post hoc analysis in patients with hypophosphatemia (<0.7 mmol/L) showed that this was frequently accompanied by other types of tubular dysfunction in CCS but not in controls (Table S1).

LMWP was more often seen in CCS (20.1%) than controls (0.4%). Low serum bicarbonate levels were found in 26 CCS (2.5%). None of them received supplementation.

CCS more often had a combination of tubular dysfunctions compared to controls, *p* <0.001 (Table 4). Moreover, LMWP and renal magnesium loss were associated with decreased GFR stages and albuminuria among CCS (Table 5). This association was not seen in controls.

### 3.3. Risk Factors for Tubular Dysfunction

#### 3.3.1. Risk Factors in CCS Compared to Controls

**Figure 2:**
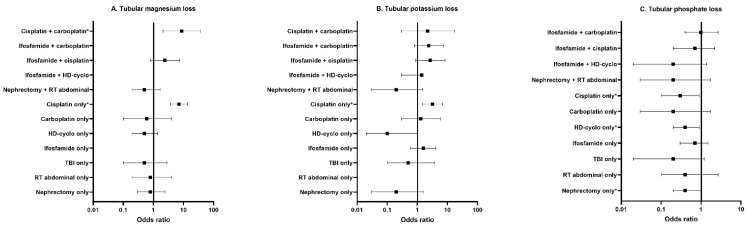
Multivariable logistic regression analyses including mutually exclusive treatment groups for tubular outcomes including: (**A**) tubular magnesium loss; (**B**) tubular potassium loss; (**C**) tubular phosphate loss. This figure displays the odds ratios in CCS compared to controls. Exact values of the odds ratios are listed in Table S1. The square represents the odds ratio, and the horizontal lines represent the 95% confidence interval. The vertical line represents the value 1 (no difference between CCS and controls). The model for tubular magnesium loss is corrected for age at study, estimated glomerular filtration rate and albumin-to-creatinine ratio. The models for tubular potassium loss are corrected for age at study. The model for tubular phosphate loss is corrected for age at study and sex. * = *p*-value < 0.05. Abbreviations: CCS, childhood cancer survivors; HD-cyclo, high-dose cyclophosphamide; RT, radiotherapy; TBI, total body irradiation.

In Figure 2 and Table S2, the prevalence and odds ratios of mutually exclusive treatment groups compared to controls are presented.

The prevalence and odds ratio for magnesium loss were significantly increased for CCS treated with cisplatin only (25.8%, OR 7.1, 95% CI 3.7–13.7) or in combination with carboplatin (23.1%, OR 8.7, 95% CI 2.1–36.3). CCS exposed to cisplatin only were also at risk for potassium loss in comparison with controls (prevalence 12%, OR 3.2, 95% CI 1.5–7.0). For tubular phosphate loss, CCS showed no increased risk compared to controls. Last, all assessed treatment groups had a higher prevalence of LMWP than controls. Multivariable risk analyses could not be performed for LMWP due to the low prevalence in controls.

#### 3.3.2. Tumor Type

**Figure 3:**
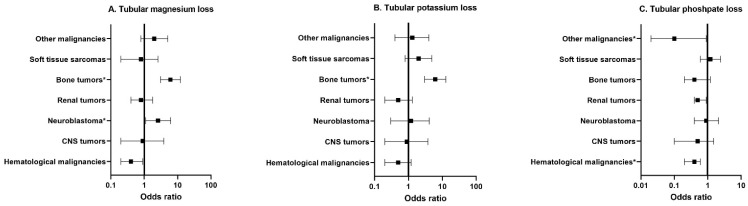
Multivariable logistic regression analyses among different tumor types for tubular outcomes including: (**A**) tubular magnesium loss; (**B**) tubular potassium loss; (**C**) tubular phosphate loss. This figure displays the odds ratios in CCS compared to controls. Exact values of the odds ratios are listed in Table S2. The square represents the odds ratio, and the horizontal lines represent the 95% confidence interval. The vertical line represents the value 1 (no difference between CCS and controls). The model for tubular magnesium loss is corrected for age at study, estimated glomerular filtration rate and albumin-to-creatinine ratio. The model for tubular potassium loss is corrected for age at study. The model for tubular phosphate loss is corrected for age at study and sex. * = *p*-value < 0.05. Abbreviations: CCS, childhood cancer survivors; CNS, central nervous system.

As a consequence of the chemotherapeutic agents used in the respective treatment protocols, survivors of bone tumors had increased odds for tubular magnesium loss (OR 6.1, 95% CI 3.1–12.1) and tubular potassium loss (OR 6.2, 95% CI 3.0–12.7) (Figure 3, Table S3). Higher odds ratios for tubular magnesium loss were also observed for survivors of neuroblastoma (OR 2.6, 95% CI 1.1–6.2).

#### 3.3.3. Risk Factors among CCS

The results of the multivariable logistic regression analyses for the tubular outcomes among CCS are presented in Table 6.

Cisplatin was associated with tubular magnesium loss (OR 10.5, 95% CI 4.1–27.2). This association was significant for all doses but highest for a cumulative dose >500 mg/m^2^ (OR 22.0, 95% CI 7.2–67.3).

Treatment risk factors significantly associated with tubular potassium loss were ifosfamide (OR 2.4, 95% CI 1.2–4.7) and cisplatin (OR 3.5, 95% CI 1.6–7.5). For ifosfamide, this association was independent of dose. Cisplatin increased the odds for potassium loss only for a cumulative dose >500 mg/m^2^ (OR 17.7, 95% CI 6.2–50.4). Carboplatin was not associated with tubular potassium loss when analyzed as a dichotomous variable but showed an increased OR for a cumulative dose > 2800 mg/m^2^ (OR 5.1, 95% CI 1.7–15.8).

Ifosfamide treatment was associated with tubular phosphate loss (OR 2.3, 95% CI 1.2–4.3), in particular for CCS exposed to >42 g/m^2^ (OR 4.1, 95% CI 1.6–10.4). CCS treated with a cumulative cisplatin dose > 500 mg/m^2^ had increased odds as well (OR 3.6, 95% CI 1.2–10.9).

The odds for LMWP were increased by ifosfamide (OR 2.2, 95% CI 1.5–3.3), especially after cumulative doses of 12–42 g/m^2^ (OR 2.0, 95% CI 1.1–3.6) and >42 g/m^2^ (OR 6.2, 95% CI 3.4–11.5).

No treatment-related risk factors were identified for decreased serum bicarbonate levels. A longer follow-up period was associated with phosphate loss but not with other tubular outcomes.

## 4. Discussion

This study assessed the prevalence of and risk factors for tubular dysfunction in a nationwide cohort of very long-term CCS treated with potentially nephrotoxic therapy in comparison with matched controls. We found a high prevalence of LMWP in CCS. The prevalence of decreased serum levels of electrolytes was not more common in CCS compared to controls, yet several CCS used electrolyte supplementation which was not the case in controls.

The high prevalence (20%) of LMWP in CCS is a strong indicator for chronic tubular damage. α1MG has proven to be the most valuable in the early detection of acute and chronic tubular injury because of its lower pre-renal variability and high stability in urine [32]. Decreased reabsorption of α1MG has only been evaluated in childhood survivors of HSCT. A prevalence of 39% was described 2 years after transplant, and no risk factors were identified [33]. We showed that this risk was particularly increased after ifosfamide exposure. Recently, increased α1MG in the urine has been reported to be a predictor of chronic kidney disease (CKD) progression and higher mortality [34], probably reflecting tubulointerstitial damage [35]. Although we could not investigate the prognostic value of α1MG because of the cross-sectional design, a strong association of α1MG with decreased GFR and albuminuria was observed. More research regarding α1MG as an early marker of renal dysfunction in CCS is needed. For now, closer surveillance might be considered in CCS with an abnormal α1MG/creatinine index. It should be borne in mind that LMWP and microalbuminuria are missed by urinary dipstick analysis [35].

Consistent with two previous studies using multivariable regression analyses, we identified cisplatin as a risk factor for tubular magnesium loss [8,18]. Contrary to our study, Stohr et al. also found an association between carboplatin and lower magnesium levels in sarcoma survivors [8]. However, they also reported a rise in magnesium levels in the first 3 years of follow-up. This suggests reversible toxicity and might explain why we did not find carboplatin as a risk factor after prolonged follow-up. Knijnenburg et al. reported higher odds for hypomagnesemia in two mutually exclusive treatment groups of CCS: nephrectomy only and combined treatment of platinum agents and ifosfamide. However, these associations had very wide confidence intervals and were not confirmed in our study. Among CCS, we observed an association of magnesium loss with higher CKD stages and albuminuria. Hypomagnesemia has been described as a predictor for mortality and GFR decline in CKD patients [36]. Moreover, during cisplatin courses, magnesium supplementation may have a kidney-protective effect [37,38]. However, its potential as a modifiable risk factor for CKD remains to be established.

Tubular potassium loss was associated with ifosfamide, cisplatin > 500 mg/m^2^ and carboplatin > 2800 mg/m^2^. Although severe hypokalemia can cause serious adverse events such as arrhythmias, the clinical impact of chronic hypokalemia is not well understood [39].

A notable finding of our study was the higher prevalence of tubular phosphate loss in controls (10.8%) compared to CCS (5.5%). Post hoc analysis showed a comparable prevalence of 10% in the total Lifelines cohort (data not shown), excluding potential selection bias of our control group. The observed difference might be explained in part by the fact that phosphate was measured in a different clinical laboratory. However, all participating clinical laboratories used the same laboratory methods and take part in external quality assessment programs. Since the implementation of these programs, inter-laboratory variation has been significantly reduced in the Netherlands [25]. The prevalence of hypophosphatemia might be overestimated in controls since 2.5–3% of the population is expected to have values below the defined lower reference limit in case of equal distribution [25]. Secondly, more controls than CCS had fasting blood tests. In the literature, lower serum phosphate levels have been observed in individuals fasting ≥ 12 h [40]. The observed difference is difficult to elucidate and might have been influenced by a confounder we are not aware of. However, the finding that hypophosphatemia was associated with additional tubular dysfunctions in CCS but not in controls supports the hypothesis that the prevalence of hypophosphatemia in controls was overestimated.

Tubular phosphate loss was associated with exposure to ifosfamide congruent with previous studies [11,12], especially for cumulative doses > 42 g/m^2^ and cisplatin doses > 500 mg/m^2^. In CCS solely treated with ifosfamide, prevalence was 9% which is comparable with the study of Skinner et al. who reported decreased serum phosphate levels of 8% after a 10-year follow-up [10]. The association of cisplatin doses >500 mg/m^2^ with long-term phosphate loss has not been described by others. This finding may prompt that CCS exposed to HD-cisplatin should be considered for phosphate screening as well, which is not currently recommended by all screening guidelines [41].

Renal tubular acidosis is most often seen as part of renal Fanconi syndrome during and early after ifosfamide and cisplatin treatment [8,42,43]. Renal acid base handling seems to recover in CCS since decreased bicarbonate levels were rarely seen and were not associated with treatment factors in our study and others [10,12].

Tubular dysfunction was mainly observed in bone tumor survivors. This is most likely because treatment regimens of Ewing sarcoma and osteosarcoma involve high dosages of nephrotoxic agents. Children with Ewing sarcoma receive high doses of ifosfamide [15]. Cyclophosphamide has been evaluated as an alternative for ifosfamide in the consolidation of standard risk Ewing sarcoma. This resulted in less tubular dysfunction, but its non-inferiority as compared to ifosfamide is uncertain [44]. For osteosarcoma, treatment consists of methotrexate, doxorubicin and cisplatin [16]. Nephrotoxicity caused by methotrexate is reversible [18,19,45,46]. Hence, long-term toxicity is most likely caused by cisplatin. These findings may guide the development of treatment protocols and emphasize the importance of nephroprotective strategies. Please note that some ascertained tumor groups are quite heterogeneous such as hematological malignancies including different type of leukemias and lymphomas with differences in treatment protocols. The effect of single treatment modalities therefore remains most important.

Current long-term follow-up guidelines for CCS differ in their recommendations regarding tubular dysfunction screening. The Children’s Oncology Group (COG) recommends screening of all electrolytes at entry of long-term follow-up for all potential nephrotoxic therapies [13]. The United Kingdom Children’s Cancer Study group recommends phosphate and bicarbonate screening for CCS exposed to ifosfamide only and magnesium screening for CCS exposed to cisplatin or carboplatin only [47]. The Dutch Childhood Oncology group recommends electrolyte screening for all CCS treated with ifosfamide, cisplatin, bilateral renal radiation, partial bilateral nephrectomy or HSCT [14].

Recently, the International Late Effects of Childhood Cancer Guideline Harmonization Group (IGHG) was established [48]. This collaborative endeavor aims to develop evidence-based harmonized guidelines for the surveillance of chronic health problems in CCS, including nephrotoxicity. The results of our multivariable analyses can inform future guidelines. The following subgroups are identified that could benefit from tubular dysfunction screening: after ifosfamide exposure screening on hypophosphatemia and hypokalemia, after cisplatin exposure screening on hypophosphatemia, hypomagnesemia and hypokalemia and after high dose carboplatin exposure screening on hypokalemia.

The strengths of this study are the large sample size, detailed treatment information, long follow-up period, comprehensive assessment of tubular function and comparison with a matched control group. However, some limitations need to be addressed. First, some outcomes were present in relatively few survivors, thereby limiting the power of some analyses. Second, as only 54% of eligible CCS participated, selection bias cannot be completely ruled out. Third, measurements were performed in different laboratories for CCS and controls resulting in potential inter-laboratory variation. However, all laboratories participate in the Dutch External Quality Assessment program, which has been very effective in reducing inter-laboratory variation in electrolyte measurements [49]. Lastly, supportive care drugs associated with acute tubular injury [50,51,52] were not taken into account. Their effect on long-term tubular function in CCS remains unknown.

## 5. Conclusions

In conclusion, 20% of long-term CCS treated with potentially nephrotoxic therapy have LMWP, but tubular electrolyte loss is infrequent. Still, some CCS have tubular dysfunction after a median follow-up of 25 years. Ifosfamide exposure is a risk factor for potassium loss, phosphate loss (when cumulative dose > 42 g/m^2^) and LMWP. Cisplatin treatment increases the odds for magnesium loss and at cumulative doses > 500 mg/m^2^ also for potassium and phosphate loss. A carboplatin cumulative dose > 2800 mg/m^2^ is associated with potassium loss. Magnesium loss and LMWP are associated with higher stages of CKD and albuminuria. Our results emphasize the importance of monitoring tubular function in CCS exposed to ifosfamide, cisplatin and carboplatin. Future studies should further elaborate on the clinical impact of chronic tubular dysfunction in CCS and the potential of hypomagnesemia as a modifiable risk factor for glomerular function.

## Figures and Tables

**Table 1:** Reference values for TmP/GFR in adults.

Age	Male Range (mmol/L)	Female Range (mmol/L)
25–35 years	1.00–1.35	0.96–1.44
45–55 years	0.90–1.35	0.88–1.42
65–75 years	0.80–1.35	0.80–1.35

Reprinted with permission from [28]. Copyright © 1998, © SAGE Publications.

**Table 2:** Baseline characteristics study cohort.

Characteristics	Underlying Cohort	Invited Study Population	Non-Participants ^b^	Participants	Controls
	*n* = 6165	*n* = 1881	*n* = 787	*n* = 1024	*n* = 500
Sex, *n* (%)					
Male	3433 (55.7)	**1009 (53.6)**	484 (61.5)	**505 (49.3)**	241 (48.2)
Female	2731 (44.3)	**872 (46.4)**	303 (38.5)	**519 (50.7)**	259 (51.8)
Transgender	1 (0.01)	0 (0)	0 (0)	0 (0)	0 (0)
Primary childhood cancer (ICCC), *n* (%)					
Leukemias, myeloproliferative diseases and myelodysplastic diseases	2094 (34.0)	569 (30.2)	225 (28.6)	317 (31.0)	−
Lymphomas and reticuloendothelial neoplasms	1062 (17.2)	150 (8.0)	68 (8.6)	79 (7.7)	−
CNS and miscellaneous intracranial and intraspinal neoplasms	844 (13.7)	121 (6.4)	55 (7.0)	62 (6.1)	−
Neuroblastoma and other peripheral nervous cell tumors	324 (5.3)	94 (5.0)	28 (3.6)	65 (6.3)	−
Retinoblastoma	33 (0.5)	2 (0.1)	1 (0.1)	1 (0.1)	−
Renal tumors	596 (9.7)	476 (25.3)	200 (25.4)	254 (24.8)	−
Hepatic tumors	52 (0.8)	34 (1.8)	22 (2.8)	12 (1.2)	−
Bone tumors	370 (6.0)	148 (7.9)	67 (8.5)	78 (7.6)	−
Soft tissue and other extraosseous sarcomas	450 (7.3)	168 (8.9)	72 (9.1)	92 (9.0)	−
Germ cell tumors, trophoblastic tumors, and neoplasms of gonads	232 (3.8)	99 (5.3)	41 (5.2)	52 (5.1)	−
Other malignant epithelial neoplasms and malignant melanomas	102 (1.7)	18 (1.0)	8 (1.0)	10 (1.0)	−
Other and unspecified malignant neoplasms	6 (0.1)	2 (0.1)	0 (0)	2 (0.2)	−
Age at diagnosis (yr), *n* (%) *					
0–4	2727 (45.3)	994 (52.9)	417 (53.1)	537 (52.4)	−
5–9	1628 (27.1)	476 (25.3)	198 (25.2)	265 (25.9)	−
10–14	1285 (21.4)	312 (16.6)	128 (16.3)	171 (16.7)	−
15–17	376 (6.3)	98 (5.2)	43 (5.5)	51 (5.0)	−
Treatment period, *n* (%)					
1963–1969	119 (1.9)	20 (1.1)	6 (0.8)	14 (1.4)	−
1970–1979	978 (15.9)	130 (6.9)	54 (6.9)	72 (7.0)	−
1980–1989	1931 (31.3)	477 (25.4)	184 (23.4)	272 (26.6)	−
1990–1999	2541 (41.2)	1093 (58.1)	479 (60.9)	576 (56.3)	−
2000–2001	596 (9.7)	161 (8.6)	64 (8.1)	90 (8.8)	−
Age at participation/invitation (yr), *n* (%) ^#^					
<18	49 (1.2)	0 (0)	0 (0)	0 (0)	0 (0)
18–30	1313 (32.9)	640 (39.1)	205 (37.8)	381 (37.2)	182 (36.4)
30–40	1511 (37.9)	709 (43.3)	244 (45.1)	446 (43.6)	216 (43.2)
>40	1118 (28.0)	286 (17.5)	92 (17.0)	197 (19.2)	102 (20.4)
Follow-up time since childhood cancer diagnosis (yr), *n* (%)					
10–20	981 (15.9)	328 (17.4)	133 (16.9)	186 (18.2)	−
20–30	1931 (31.3)	1078 (57.3)	469 (59.6)	569 (55.6)	−
30–40	1393 (22.6)	351 (18.7)	136 (17.3)	197 (19.2)	−
40–50	460 (7.5)	112 (6.0)	48 (6.1)	61 (6.0)	−
50–60	46 (0.7)	12 (0.6)	1 (0.1)	11 (1.1)	−
Surgery, *n* (%) ^a^					
No	2912 (47.2)	694 (36.9)	281 (35.7)	385 (37.6)	−
Yes	3185 (51.7)	1182 (62.8)	503 (63.9)	637 (62.2)	−
Missing	68 (1.1)	5 (0.3)	3 (0.4)	2 (0.2)	−
Radiotherapy, *n* (%) ^a^					
No	3608 (58.5)	**1177 (62.6)**	533 (67.7)	**596 (58.2)**	−
Yes	2527 (41.0)	**703 (37.4)**	254 (32.3)	**427 (41.7)**	−
Missing	30 (0.5)	1 (0.05)	0 (0)	1 (0.1)	−
Chemotherapy, *n* (%) ^a^					
No	1123 (18.2)	35 (1.9)	15 (1.9)	20 (2.0)	−
Yes	5005 (81.2)	1845 (98.1)	771 (98.0)	1004 (98.0)	−
Missing	37 (0.6)	1 (0.05)	1 (0.1)	0 (0)	−
Stem cell transplantation/reinfusion, *n* (%) ^a,^*					
No	5532 (89.7)	1624 (86.4)	698 (88.8)	863 (84.3)	−
Autologous transplant	155 (2.5)	90 (4.8)	34 (4.3)	56 (5.5)	−
Allogeneic HSCT	231 (3.7)	153 (8.1)	51 (6.5)	95 (9.3)	−
Missing	98 (1.6)	13 (0.7)	3 (0.4)	10 (1.0)	−
Therapy, *n* (%)					
No treatment	61 (1.0)	0 (0)	0 (0)	0 (0)	−
Surgery only	575 (9.3)	17 (0.9)	8 (1.0)	9 (0.9)	−
Chemotherapy only (±surgery)	2967 (48.1)	1160 (61.7)	525 (66.7)	587 (57.3)	−
Radiotherapy only (±surgery)	484 (7.9)	18 (1.0)	7 (0.9)	11 (1.1)	−
Chemotherapy and radiotherapy (±surgery)	2030 (32.9)	684 (36.4)	246 (31.3)	416 (40.6)	−
Missing	48 (0.8)	2 (0.1)	1 (0.1)	1 (0.1)	−
Potentially nephrotoxic cancer treatment, *n* (%) ^a^					
Nephrectomy	622 (10.1)	492 (26.2)	207 (26.3)	264 (25.8)	−
Unilateral	605 (97.3)	478 (97.2)	204 (98.6)	255 (96.6)	−
Bilateral partial	17 (2.7)	14 (2.9)	3 (1.5)	9 (3.4)	−
Radiotherapy renal area	467 (7.6)	273 (14.5)	90 (11.4)	175 (17.1)	−
Total body irradiation	221 (3.6)	143 (7.6)	52 (6.6)	85 (8.4)	−
Ifosfamide	714 (11.6)	524 (27.9)	206 (26.2)	300 (29.3)	−
HD-cyclophosphamide	833 (13.5)	504 (26.8)	208 (26.4)	278 (27.2)	−
Cisplatin	457 (7.4)	328 (17.4)	142 (18.0)	175 (17.1)	−
Carboplatin	422 (6.9)	284 (15.1)	125 (15.9)	151 (14.7)	−
Allogeneic HSCT	231 (3.8)	153 (8.1)	51 (6.5)	95 (9.3)	−

^a^ For primary cancer and recurrences. ^b^ Non-participants includes refusers and non-responders. CCS with informed consent without participation (*n* = 53), being pregnant (*n* = 8) or having a history of kidney transplantation (*n* = 9) were not included in this table because they were willing to participate. * Missing for survivors refusing registration, *n* = 149. ^#^ Missing for survivors refusing participation, *n* = 2174. Bold = *p*-value < 0.05. Abbreviations: HD, high dose; HSCT, hematopoietic stem cell transplantation; *n*, number; yr, year.

**Table 3:** Prevalence of tubular dysfunction in childhood cancer survivors compared to matched controls.

Tubular Function Parameter	CCS (*n*)	Prevalence ^a^	Controls (*n*)	Prevalence ^a^	*p*-Value
Tubular magnesium loss	999	56/999 (5.6)	500	25/500 (5.0)	0.63
Magnesium supplementation	1024	20/1024 (2.0)	500	0/500 (0)	<0.001
Tubular potassium loss	1003	45/1003 (4.5)	500	20/500 (4.0)	0.66
Potassium supplementation	1024	9/1024 (0.9%)	500	0/500 (0)	0.04
Tubular phosphate loss	997	55/997 (5.5)	500	54/500 (10.8)	<0.001
Phosphate supplementation	1024	3/1024 (0.3%)	500	0/500 (0)	0.55
Low molecular weight proteinuria	931	187/931 (20.1)	498	2/498 (0.4)	<0.001
Metabolic acidosis	1002	26/1002 (2.5)	-	-	-

^a^ Values are the number of participants with a positive test result/total number of participants tested (percentage). Abbreviations: CCS, childhood cancer survivors; *n*, number.

**Table 4:** Total number of tubular outcomes in childhood cancer survivors and matched controls.

Total Number of Tubular Outcomes	CCS (*n*)	Controls (*n*)
0	658 (71.1)	401 (80.5)
1	213 (23.0)	93 (18.7)
2	45 (4.9)	4 (0.8)
3	9 (1.0)	0 (0)
4	1 (0.1)	0 (0)

Abbreviations: CCS, childhood cancer survivors; *n*, number.

**Table 5:** Relation between glomerular function and tubular outcomes in childhood cancer survivors and controls.

	LMWP		Renal Magnesium Loss		Renal Potassium Loss		Renal Phosphate Loss	
	CCS	Controls	CCS	Controls	CCS	Controls	CCS	Controls
G1 (eGFR ≥ 90)	**111/692 (16.0)**	2/427 (0.5)	**34/715 (4.8)**	25/429 (5.8)	26/717 (3.6)	19/429 (4.6)	36/713 (5.0)	48/429 (11.2)
G2 (eGFR 60–89)	**51/185 (27.6)**	0/71 (0.0)	**18/189 (9.5)**	0/71 (0.0)	15/191 (7.9)	1/71 (1.4)	17/190 (8.9)	6/71 (8.5)
G3 (eGFR 45–59)	**14/21 (66.7)**	0/0 (0.0)	**2/21 (9.5)**	0/0 (0.0)	2/21 (9.5)	0/0 (0.0)	1/21 (4.8)	0/0 (0.0)
G4 (eGFR 15–44)	**3/3 (100)**	0/0 (0.0)	**2/4 (50.0)**	0/0 (0.0)	0/4 (0.0)	0/0 (0.0)	1/4 (25.0)	0/0 (0.0)
G5 (eGFR < 15)	**1/1 (100)**	0.0 (0.0)	**0/1 (0.0)**	0.0 (0.0)	0/1 (0.0)	0.0 (0.0)	0/1 (0.0)	0.0 (0.0)
No albuminuria	**112/756 (14.8)**	2/492 (0.4)	**37/775 (4.8)**	25/494 (5.1)	32/776 (4.1)	20/494 (4.0)	41/775 (5.3)	53/494 (10.7)
Microalbuminuria	**60/138 (43.5)**	0/5 (0.0)	**13/140 (9.3)**	0/5 (0.0)	12/142 (8.5)	0/5 (0.0)	13/142 (9.2)	1/5 (20.0)
Macroalbuminuria	**6/10 (60.0)**	0/1 (0.0)	**4/10 (40.0)**	0/1 (0.0)	1/10 (10.0)	0/1 (0.0)	0/10 (0.0)	0/1 (0.0)

Values are the number of participants with a positive test result/total number of participants tested (percentage). Bold = *p*-value < 0.05. Abbreviations: CCS, childhood cancer survivors; eGFR, estimated glomerular filtration rate in mL/min/1.73 m^2^; LMWP, low molecular weight proteinuria.

**Table 6:** Multivariable logistic regression analyses for tubular outcomes in childhood cancer survivors including independent treatment variables.

	**Tubular Magnesium Loss** ***n*** **= 56/999**		**Tubular Potassium Loss** ***n*** **= 43/1003**			**Tubular Phosphate Loss** ***n*** **= 55/997**			**Low Molecular Weight Proteinuria** ***n*** **= 187/931**			
**Model 1**	**Prevalence ^a^**	**OR (95% CI)** **Multivariable**		**Prevalence ^a^**	**OR (95% CI)** **Multivariable**		**Prevalence ^a^**	**OR (95% CI)** **Multivariable**		**Prevalence ^a^**	**OR (95% CI)** **Multivariable**	
**Nephrectomy**												
No	46/738 (6.2)	1.0 (ref)		40/741 (5.4)	1.0 (ref)		42/736 (5.7)	1.0 (ref)		149/687 (21.7)	1.0 (ref)	
Yes	10/261 (3.8)	0.9 (0.3–2.7)		5/262 (1.9)	0.6 (0.2–2.1)		13/261 (5.0)	1.2 (0.5–2.9)		38/244 (15.6)	0.5 (0.3–0.8)	
**Abdominal RT**												
No	45/811 (5.5)	1.0 (ref)		35/814 (4.3)	1.0 (ref)		42/808 (5.2)	1.0 (ref)		152/754 (20.2)	1.0 (ref)	
Yes	9/173 (5.2)	1.1 (0.4–2.8)		7/174 (4.0)	1.9 (0.7–5.2)		10/174 (5.7)	1.3 (0.5–3.0)		32/162 (19.8)	1.1 (0.6–2.0)	
**TBI**												
No	52/902 (5.8)	1.0 (ref)		40/904 (4.4)	1.0 (ref)		48/898 (5.3)	1.0 (ref)		166/837 (19.8)	1.0 (ref)	
Yes	2/82 (2.4)	0.8 (01.1–4.2)		2/84 (2.4)	0.8 (0.2–3.8)		4/84 (4.8)	1.0 (0.3–3.0)		18/79 (22.8)	1.1 (0.6–2.1)	
**Ifosfamide**												
No	49/703 (7.0)	1.0 (ref)		21/706 (3.0)	1.0 (ref)		28/702 (4.0)	**1.0 (ref)**		92/652 (14.1)	1.0 (ref)	
Yes	7/296 (2.4)	0.2 (0.1–0.6)		24/297 (8.1)	**2.4 (1.2–4.7)**		27/295 (9.2)	**2.3 (1.2–4.3)**		95/279 (34.1)	**2.2 (1.5–3.3)**	
**HD-cyclo**												
No	52/731 (7.1)	1.0 (ref)		41/734 (5.6)	1.0 (ref)		44/731 (6.0)	1.0 (ref)		148/685 (21.6)	1.0 (ref)	
Yes	4/266 (1.5)	0.5 (0.1–1.7)		4/267 (1.5)	0.5 (0.1–1.5)		11/264 (4.2)	0.8 (0.4–1.9)		39/244 (16.0)	0.7 (0.4–1.2)	
**Cisplatin**												
No	20/829 (2.4)	1.0 (ref)		26/832 (3.1)	1.0 (ref)		45/826 (5.4)	1.0 (ref)		156/771 (20.2)	1.0 (ref)	
Yes	36/170 (21.2)	**10.5 (4.1–27.2)**		19/171 (11.1)	**3.5 (1.6–7.5)**		10/171 (5.8)	1.2 (0.5–2.8)		31/160 (19.4)	0.8 (0.5–1.3)	
**Carboplatin**												
No	49/852 (5.8)	1.0 (ref)		33/855 (3.9)	1.0 (ref)		42/849 (4.9)	1.0 (ref)		149/790 (18.9)	1.0 (ref)	
Yes	7/147 (4.8)	1.1 (0.4–3.3)		12/148 (8.1)	1.6 (0.7–3.8)		13/148 (8.8)	1.5 (0.7–3.3)		38/141 (27.0)	1.3 (0.8–2.1)	
**HSCT**												
No	51/899 (5.7)	NA		42/903 (4.7)	NA		50/897 (5.6)	NA		164/838 (19.6)	NA	
Yes	2/91 (2.2)			2/91 (2.2)			4/91 (4.4)			19/84 (22.6)		
**Gender**												
Male	21/492 (4.3)	NA		19/496 (3.8)	NA		29/492 (5.9)	NA		91/461 (19.7)	NA	
Female	35/507 (6.9)			26/507 (5.1)			26/505 (5.1)			96/470 (20.4)		
**Age at diagnosis**	-	1.0 (0.96–1.1)		-	1.1 (0.99–1.1)			NA		-	NA	
**FU duration (yr)**												
10–19	11/181 (6.1)	1.0 (ref)		12/181 (6.6)	1.0 (ref)		4/180 (2.2)	1.0 (ref)		37/162 (22.8)	1.0 (ref)	
20–29	20/554 (3.6)	0.9 (0.4–2.2)		23/555 (4.1)	0.9 (0.4–1.9)		38/550 (6.9)	4.7 (1.4–15.5)		96/513 (18.7)	0.9 (0.6–1.6)	
≥30	25/264 (9.5)	1.3 (0.5–3.4)		10/267 (3.7)	0.8 (0.3–2.0)		13/267 (4.9)	3.3 (0.9–12.5)		54/256 (21.1)	0.9 (0.5–1.5)	
**eGFR (per 1 mL/min/1.73 m^2^)**		**0.98 (0.96–0.99)**									**0.98 (0.96–0.99)**	
**ACR (per 1 mg/mmol)**		1.0 (0.9–1.02)									**1.06 (1.02–1.09)**	
**Model 2**	**Prevalence ^a^**	**OR (95% CI)** **Multivariable**	P_trend_ *	**Prevalence^a^**	**OR (95% CI)** **Multivariable**	P_trend_ *	**Prevalence ^a^**	**OR (95% CI)** **Multivariable**	P_trend_ *	**Prevalence ^a^**	**OR (95% CI)** **Multivariable**	P_trend_ *
**Abdominal RT dose, Gy**												
None							42/808 (5.2)	1.0 (ref)		152/754 (20.2)	1.0 (ref)	
<20							3/47 (6.4)	1.6 (0.4–6.4)		8/43 (18.6)	1.6 (0.6–3.9)	
20–30							2/54 (3.7)	0.9 (0.2–4.5)		9/50 (18.0)	1.3 (0.5–3.2)	
>30							5/71 (7.0)	1.4 (0.5–3.9)	0.66	15/67 (22.4)	1.0 (0.5–2.1)	0.95
**Ifosfamide dose, mg/m^2^**												
None				21/706 (3.0)	1.0 (ref)		28/702 (4.0)	1.0 (ref)		92/652 (14.1)	1.0 (ref)	
≤12,000				5/99 (5.1)	**3.7 (1.2–11.7)**		6/99 (6.1)	1.6 (0.6–4.5)		17/91 (18.7)	1.1 (0.6–2.2)	
12,001–42,000				9/97 (9.3)	**2.4 (0.9–6.4)**		9/97 (9.3)	2.4 (1.0–5.9)		27/92 (29.3)	**2.0 (1.1–3.6)**	
>42,000				9/99 (9.1)	**3.7 (1.3–10.7)**	0.56	12/97 (12.4)	**4.1 (1.6–10.4)**	0.39	50/94 (53.2)	**6.2 (3.4–11.5)**	0.16
**Cisplatin dose mg/m^2^**												
None	20/829 (2.4)	1.0 (ref)		26/832 (3.1)	1.0 (ref)		45/826 (5.4)	1.0 (ref)		156/771 (20.2)	1.0 (ref)	
≤300	6/57 (10.5)	**5.8 (1.7–19.9)**		2/58 (3.4)	1.0 (0.2–5.3)		2/58 (3.4)	0.8 (0.2–3.9)		12/55 (21.8)	1.1 (0.5–2.5)	
301–500	10/57 (17.5)	**9.2 (2.9–29.2)**		3/57 (5.3)	1.8 (0.4–7.5)		2/57 (3.5)	0.5 (0.1–3.6)		9/54 (16.7)	1.0 (0.4–2.3)	
>500	20/55 (36.4)	**22.0 (7.2–67.3)**	0.72	14/55 (25.5)	**17.7 (6.2–50.4)**	0.84	6/55 (10.9)	**3.6 (1.2–10.9)**	0.85	10/50 (20.0)	1.1 (0.5–2.6)	0.36
**Carboplatin dose, mg/m^2^**												
None				33/855 (3.9)	1.0 (ref)		42/849 (4.9)	1.0 (ref)		149/790 (18.9)	1.0 (ref)	
≤1500				5/51 (9.8)	1.1 (0.2–5.7)		5/51 (9.8)	1.6 (0.5–5.4)		17/49 (34.7)	1.2 (0.5–2.6)	
1501–2800				1/49 (2.0)	0.6 (0.1–5.2)		6/49 (12.2)	2.8 (1.0–7.9)		12/47 (25.5)	2.5 (1.1–5.5)	
>2800				6/46 (13.0)	**5.1 (1.7–15.8)**	**0.04**	2/46 (4.3)	0.7 (0.2–3.5)	0.74	9/43 (20.9)	0.9 (0.3–2.1)	**0.02**
**Cisplatin dose mg/m^2^**												
None	20/829 (2.4)	1.0 (ref)		26/832 (3.1)	1.0 (ref)		45/826 (5.4)	1.0 (ref)		156/771 (20.2)	1.0 (ref)	
≤300	6/57 (10.5)	**5.8 (1.7–19.9)**		2/58 (3.4)	1.0 (0.2–5.3)		2/58 (3.4)	0.8 (0.2–3.9)		12/55 (21.8)	1.1 (0.5–2.5)	
301–500	10/57 (17.5)	**9.2 (2.9–29.2)**		3/57 (5.3)	1.8 (0.4–7.5)		2/57 (3.5)	0.5 (0.1–3.6)		9/54 (16.7)	1.0 (0.4–2.3)	
>500	20/55 (36.4)	**22.0 (7.2–67.3)**	0.72	14/55 (25.5)	**17.7 (6.2–50.4)**	0.84	6/55 (10.9)	**3.6 (1.2–10.9)**	0.85	10/50 (20.0)	1.1 (0.5–2.6)	0.36
**Carboplatin dose, mg/m^2^**												
None				33/855 (3.9)	1.0 (ref)		42/849 (4.9)	1.0 (ref)		149/790 (18.9)	1.0 (ref)	
≤1500				5/51 (9.8)	1.1 (0.2–5.7)		5/51 (9.8)	1.6 (0.5–5.4)		17/49 (34.7)	1.2 (0.5–2.6)	
1501–2800				1/49 (2.0)	0.6 (0.1–5.2)		6/49 (12.2)	2.8 (1.0–7.9)		12/47 (25.5)	2.5 (1.1–5.5)	
>2800				6/46 (13.0)	**5.1 (1.7–15.8)**	**0.04**	2/46 (4.3)	0.7 (0.2–3.5)	0.74	9/43 (20.9)	0.9 (0.3–2.1)	**0.02**

All factors in Model 1 have been adjusted for simultaneously. Model 2 was similar to Model 1, except that the dichotomous treatment modalities have been substituted by cumulative doses if applicable. The other variables are not shown for Model 2 for clarity. Numbers do not always add up to the total because of missing values. ^a^ Values are the number of participants with a positive test result/total number of participants tested (percentage). * Test for trend in continuous dose variable among exposed CCS. Bold = *p*-value < 0.05. Abbreviations: 95% CI, 95% confidence interval; FU, follow-up; Gy, gray; HD, high dose; HSCT, hematopoietic stem cell transplantation; NA, not applicable; OR, odds ratio; ref, reference; RT, radiotherapy; TBI, total body irradiation; yr, years.

## Data Availability

Not applicable.

## Supplementary Materials

The following supporting information can be downloaded at: https://www.mdpi.com/article/10.3390/cancers14112754/s1, Table S1: Subgroup analysis for childhood cancer survivors and controls with hypophosphatemia (phosphate < 0.70 mmol/L); Table S2: Multivariable logistic regression analyses for tubular outcomes including mutually exclusive treatment groups; Table S3: Multivariable logistic regression analyses for tubular dysfunction among different tumor types.
