# Supplementary material for: Long-Term Tubular Dysfunction in Childhood Cancer Survivors; DCCSS-LATER 2 Renal Study
Source: Cancers (Basel). 2022 Jun 1;14(11):2754. doi: 10.3390/cancers14112754 (PMC9179377; doi:10.3390/cancers14112754)
Supplement: Supplementary file 1 [file cancers-14-02754-s001.zip › cancers-1703576-supplementary.pdf]

cancers

MDPI

Long-Term Tubular Dysfunction in Childhood Cancer Survivors; DCCSS-LATER 2 Renal Study

Esmee C.M. Kooijmans*, Helena J. H. van der Pal, Saskia M. F. Pluijm,Margriet van der Heiden-van der Loo, Leontien C. M. Kremer, Dorine Bresters,Eline van Dulmen-den Broeder, Marry M. van den Heuvel-Eibrink, Jacqueline J. Loonen, Marloes Louwerens, Sebastian J. C. Neggers, Cécile Ronckers, Wim J. E. Tissing, Andrica C. H. de Vries, Gertjan J. L. Kaspers, Arend Bökenkamp† and Margreet A. Veening† on behalf of the Dutch LATER study group

Supplemental material

Table S1. Subgroup analysis for childhood cancer survivors and controls with hypophosphatemia (phosphate <0.70 mmol/L).

	CCS		Controls		
**Renal Function Parameter**	**Prevalence Subgroup**[a]	**Prevalence Total**[a]	**Prevalence Subgroup**[a]	**Prevalence Total**[a]	***p*-value***
Tubular magnesium loss	10/60 (16.7)	56/999 (5.6)	1/54 (1.9)	25/500 (5.0)	0.007
Tubular potassium loss	9/60 (15.0)	45/1003 (4.5)	2/54 (3.7)	20/500 (4.0)	0.04
Low molecular weight proteinuria	14/54 (25.9)	187/931 (20.1)	0/54 (0)	2/498 (0.4)	< 0.001

[a]Values are the number of participants with a positive test result/total number of participants tested (percentage).* p-value provided for subgroup-analyses CCS compared to controls. Abbreviations: CCS, childhood cancer survivors.

Table S2. Multivariable logistic regression analyses for tubular outcomes including mutually exclusive treatment groups.

	Tubular Magnesium Loss*		Tubular Potassium Loss+		Tubular Phosphate Loss^		Low Molecular Weight Proteinuria	
	**Prevalence**[a]	**OR (95% CI) Multivariable**	**Prevalence**[a]	**OR (95% CI) Multivariable**	**Prevalence**[a]	**OR (95% CI) Multivariable**	**Prevalence**[a]	**Fisher exact test Treatment groups vs. controls**
Controls	25/500 (5.0)	1.0 (ref)	20/500 (4.0)	1.0 (ref)	54/500 (10.8)	1.0 (ref)	2/500 (0.4)	
Nephrectomy only	6/124 (4.8)	0.8 (0.3–2.4)	1/124 (0.8)	0.2 (0.03–1.6)	5/123 (4.1)	**0.4 (0.2–0.99)**	14/115 (12.2)	$p < 0.001$
RT abdominal only	2/25 (8.0)	0.8 (0.2–4.1)	0/25 (0)	-	1/25 (4.0)	0.4 (0.1–2.7)	4/24 (16.7)	$p < 0.001$
TBI only	2/44 (4.5)	0.5 (0.1–2.8)	1/45 (2.2)	0.5 (0.1–3.8)	1/45 (2.2)	0.2 (0.02–1.2)	9/42 (21.4)	$p < 0.001$
Ifosfamide only	0/92 (0)	–	6/92 (6.5)	1.6 (0.6–4.2)	8/90 (8.9)	0.7 (0.3–1.5)	29/87 (33.3)	$p < 0.001$
HD-cyclo only	4/183 (2.2)	0.5 (0.2–1.4)	1/183 (0.5)	0.1 (0.02–1.04)	9/180 (5.0)	**0.4 (0.2–0.9)**	18/166 (10.8)	$p < 0.001$
Cisplatin only	23/89 (25.8)	**7.1 (3.7–13.7)**	11/89 (12.4)	**3.2 (1.5–7.0)**	3/89 (3.4)	**0.3 (0.1–0.9)**	11/81 (13.6)	$p < 0.001$
Carboplatin only	1/43 (2.3)	0.6 (0.1–4.1)	2/43 (4.7)	1.3 (0.3–5.8)	1/43 (2.3)	0.2 (0.03–1.7)	7/41 (17.1)	$p < 0.001$
Nephrectomy + RT abdominal	4/102 (3.9)	0.5 (0.2–1.7)	1/103 (1.0)	0.2 (0.03–1.5)	4/103 (3.9)	0.3 (0.1–0.99)	16/95 (16.8)	$p < 0.001$
Ifosfamide + HD-cyclo	0/35 (0)	-	2/35 (5.7)	1.4 (0.3–6.4)	1/35 (2.9)	0.2 (0.02–1.4)	9/31 (29.0)	$p < 0.001$
Ifosfamide + cisplatin	4/38 (10.5)	2.4 (0.8–7.5)	4/38 (10.5)	2.7 (0.9–8.4)	3/38 (7.9)	0.7 (0.2–2.2)	10/36 (27.8)	$p < 0.001$
Ifosfamide + carboplatin	0/51 (0)	-	4/51 (7.8)	2.4 (0.8–7.3)	5/51 (9.8)	0.99 (0.4–2.7)	19/48 (39.6)	$p < 0.001$
Cisplatin + carboplatin	3/13 (23.1)	**8.7 (2.1–36.3)**	1/14 (7.1)	2.2 (0.3–17.6)	0/14 (0)	NA	3/13 (23.1)	$p < 0.001$

[a]Values are the number of participants with a positive test result/total number of participants tested (percentage).* Model corrected for age at study, estimated glomerular filtration rate and albumin-creatinine ratio. + Model corrected for age at study. ^ Model corrected for age at study and sex. Bold= *p*-value < 0.05. Abbreviations: 95% CI, 95% confidence interval; HD, high-dose; NA, not applicable; OR, odds ratio; ref, reference; RT, radiotherapy; TBI, total body irradiation.

Table S3. Multivariable logistic regression analyses for tubular dysfunction among different tumor types.

	**Tubular Magnesium Loss***		**Tubular Potassium Loss+**		**Tubular Phosphate Loss^**		**Low Molecular Weight Proteinuria**	
	**Prevalence[a]**	**OR (95% CI) Multivariable**	**Prevalence[a]**	**OR (95% CI) Multivariable**	**Prevalence[a]**	**OR (95% CI) Multivariable**	**Prevalence[a]**	**Fisher Exact Test Tumor type vs. Controls**
Controls	25/500 (5.0)	1.0 (ref)	20/500 (4.0)	1.0 (ref)	54/500 (10.8)	1.0 (ref)	2/500 (0.4)	
Hematological malignancies	8/383 (2.1)	0.4 (0.2–0.9)	8/385 (2.1)	0.5 (0.2–1.2)	17/382 (4.5)	0.4 (0.2–0.6)	50/350 (14.3)	p <0.001
CNS tumors	2/57 (3.5)	0.9 (0.2–3.9)	2/57 (3.5)	0.9 (0.2–3.8)	3/57 (5.3)	0.5 (0.1–1.5)	13/54 (24.1)	p <0.001
Neuroblastoma	7/64 (10.9)	**2.6 (1.1–6.2)**	3/64 (4.7)	1.2 (0.3–4.1)	6/64 (9.4)	0.9 (0.4–2.1)	15/61 (24.6)	p <0.001
Renal tumors	10/251 (4.0)	0.8 (0.4–1.8)	5/252 (2.0)	0.5 (0. –1.3)	12/251 (4.8)	0.5 (0.4–0.9)	37/235 (15.7)	p <0.001
Bone tumors	19/77 (24.7)	**6.1 (3.1–12.1)**	16/78 (20.5)	**6.2 (3.0–12.7)**	4/76 (5.3)	0.4 (0.2–1.2)	22/71 (31.0)	p <0.001
Soft tissue sarcomas	3/91 (3.3)	0.8 (0.2–2.6)	7/91 (7.7)	2.0 (0.8–4.9)	12/91 (13.2)	1.2 (0.6–2.4)	37/89 (41.6)	p <0.001
Other malignancies	7/76 (9.2)	2.0 (0.8–5.1)	4/76 (5.3)	1.3 (0.4–4.0)	1/76 (1.3)	0.1 (0.02–0.9)	13/71 (18.3)	p <0.001

[a]Values are the number of participants with a positive test result/total number of participants tested (percentage).* Model corrected for age at study, estimated glomerular filtration rate and albumin-creatinine ratio.+ Model corrected for age at study.^ Model corrected for age at study and sex.Bold= p-value < 0.05.Abbreviations: 95% CI, 95% confidence interval; CNS, central nervous system; OR, odds ratio; ref, reference.
